# Personality, dietary identity, mental and sleep health in vegans and vegetarians: A preliminary cross‐sectional study

**DOI:** 10.1002/hsr2.1525

**Published:** 2023-08-22

**Authors:** Christle Coxon, Piril Hepsomali, Karen Brandt, David Vauzour, Adele Costabile

**Affiliations:** ^1^ School of Psychology University of Roehampton London UK; ^2^ Department of Nutrition and Preventive Medicine Norwich Medical School, University of East Anglia Norwich UK; ^3^ School of Life and Health Sciences University of Roehampton London UK

**Keywords:** anxiety, depression, nutrition, sleep quality, stress

## Abstract

**Background and Aims:**

Plant‐based diets have gained popularity over the past decade. However, research regarding mental and sleep health benefits of following plant‐based diets are conflicting. As there are associations between mental/sleep health and various personality traits, and personality may differ between individuals who follow different diets, in this preliminary study, we examined the associations between mental and sleep health and (i) personality and (ii) dietary identity in individuals who follow vegan and vegetarian diets.

**Methods:**

Cross‐sectional data on sociodemographic, personality traits, dietarian identity, overall mental health, depression, anxiety, stress, and sleep quality were collected from 57 vegan/vegetarian participants between the ages of 18–40.

**Results:**

After controlling for various sociodemographic and lifestyle factors, linear regression models revealed that (i) higher dietarian private regard was a significant predictor of better overall mental health, (ii) lower levels of extraversion and higher levels of empathy predicted depression, (iii) higher levels of neuroticism and empathy predicted anxiety, (iv) higher levels of neuroticism, dietarian centrality, and neuroticism × centrality predicted stress, (v) higher levels of conscientiousness, lower levels of dietarian centrality, but higher levels of personal motivation and dietary strictness, as well as conscientiousness × centrality, conscientiousness × personal motivation, and conscientiousness × strictness predicted better sleep quality.

**Conclusions:**

These preliminary findings suggest that not only personality traits, but also dietary identity was indeed related to mental and sleep health in individuals who follow plant‐based diets.

## Key points

What's known: ‐Plant‐based diets have gained popularity in the past decade. ‐Research on the mental and sleep health benefits of plant‐based diets is conflicting. What's new: ‐This preliminary study examined the associations between mental and sleep health and personality traits and dietary identity in individuals who follow vegan and vegetarian diets. ‐We found personality traits such as lower levels of extraversion and higher levels of empathy predicted depression, while higher levels of neuroticism and empathy predicted anxiety. ‐Higher levels of conscientiousness, personal motivation, and dietary strictness predicted better sleep quality. Clinical implications: ‐These preliminary findings suggest that both personality traits and dietary identity are related to mental and sleep health in individuals who follow plant‐based diets.

## 1 INTRODUCTION

Vegan and vegetarian diets have gained increased popularity over the past decade, and in the United Kingdom, an increased proportion of individuals report consuming plant‐based dietary patterns.^1^ While vegan and vegetarian diets are associated with positive health outcomes, including a lower body mass index (BMI), better cardiometabolic profile^2^, ^3^ and reduced risk of cardiovascular disease,^4^ research evidence suggests that individuals may be at risk of suboptimal nutrition.^5^ On the other hand, results in literature in relation to mental and sleep health are conflicting. While some investigations found positive results between vegan and/or vegetarian diets and mental and sleep health outcomes^6^, ^7^, ^8^ others reported null or inverse associations.^7^, ^8^, ^9^, ^10^, ^11^ Limitations may include inconsistencies in the definition of vegan and vegetarian diets, classification of meat products and processing^10^ or different covariates included in the statistical models. An important consideration is that psychological and behavioral characteristics may differ between vegans, vegetarians, and omnivores, therefore, variation in personality and dietary identity factors may have an impact on the associations between diet, mental and sleep health.

Personality traits are found to vary with dietary status^12^ and types of meat consumed,^13^ such that higher scores of Openness, Conscientiousness, and Agreeableness are associated with lower meat consumption. Compared to omnivores, vegetarianism is associated with higher scores of Openness and Empathy^14^, ^15^ and higher levels scores of Openness and Agreeableness.^16^ However, compared to omnivores, vegetarians were also found to score higher on neuroticism and depression.^17^ Furthermore, systematic differences in psychological traits may exist between vegans and vegetarians. One study comparing vegans and vegetarians reported that neuroticism was marginally higher in vegetarians, yet no differences were found in the other four personality domains.^18^ However, another study reported that vegans scored lower on neuroticism and higher on openness and empathy compared to vegetarians.^15^

The variation in personality traits may be linked to mental and sleep health outcomes. Research has shown that neuroticism is strongly linked to internalizing psychopathologies such as depression and anxiety disorders.^19^ Additionally, higher levels of neuroticism were found to be associated with affective disorders such as major depressive disorder and social anxiety^20^ and higher levels of self‐reported depression and anxiety^21^ while higher scores of neuroticism, and lower scores of extraversion, conscientiousness, and agreeableness, predicted an increased risk for anxiety and depression.^22^ Further longitudinal research indicates that high levels of neuroticism predict the risk for the development of anxiety and depressive disorders,^23^ and another study reported that high neuroticism and low extraversion were found to predict the chronicity of diagnoses and symptoms of various affective disorders.^24^ In addition to big five personality traits, meta‐analytic evidence showed that affective empathy (but not cognitive empathy) trait was positively correlated with depression^25^ and social anxiety.^26^

In terms of sleep, personality traits, such as neuroticism, is also shown to be associated with sleep health. Higher neuroticism was found to be correlated with poor sleep, whereas higher extraversion, agreeableness, and conscientiousness were shown to be linked to better sleep outcomes.^27^, ^28^ In line with this, high neuroticism and low conscientiousness was shown to predict poor sleep (poor sleep hygiene, low sleep quality, and increased sleepiness).^29^ Additionally, longitudinal evidence from four prospective studies has showed that high neuroticism and low extraversion were associated with worse sleep quality over time and low conscientiousness was associated with worsening of sleep quality over time.^30^

Alongside personality traits, it has been proposed that veganism extends beyond the diet, but encompasses a set of beliefs, attitudes, and motivations to which the individual identifies, and identity may shape the way in which the individual behaves/thinks/feels and communicates with the world.^31^ Accordingly, albeit limited, research has shown that vegetarians (compared to omnivores) adhered to their diets more strictly (higher strictness), felt stronger motivations to follow their diet (higher motivation), evaluated vegetarians more favorably (higher private regard), evaluated other individuals who follow other types of diets more negatively (lower out‐group regard), and felt that vegetarians were judged more negatively by others (lower public regard).^32^, ^33^, ^34^ On the other hand, omnivores evaluated individuals who follow other types of diets more positively (higher out‐group regard) and did not feel judged negatively more by other people for their dietary choices (higher public regard).^32^ Additionally, it has also been shown that vegetarians and vegans exhibit different dietarian identity profiles. For instance, it has been reported that vegans (compared to vegetarians) saw their diet as a fundamental part of their identity (higher centrality), had more positive feelings toward vegans (higher private regard), felt judged negatively more by other people for their dietary choices (lower public regard), evaluated individuals who follow other types of diets more negatively (lower out‐group regard), and had stronger motivations to adhere to veganism (higher prosocial, personal, and moral motivations).^33^, ^34^ The observed variations in identity, attitudes, and motivations associated with veganism and vegetarianism could potentially impact mental and sleep health outcomes.

Taken together, differences in personality traits and dietary identity observed in individuals who follow a vegan and vegetarian diet suggests a potential link between these factors and mental and sleep health. To the best of our knowledge, irrespective of diet type, dietary identity in relation to mental and sleep health has not yet been examined. Given the associations reported above between (i) diet type and dietary identity, personality, and mental and sleep health, as well as (ii) personality, and mental and sleep health, we expect dietary identity to contribute to the complex relationships between personality and health. Hence, in the current study, our aim was to examine the roles of personality and dietary identity in predicting mental and sleep health‐related outcomes in individuals who adhere to vegan and vegetarian diets.

## 2 METHOD

### 2.1 Study design and participant recruitment

This was a preliminary, cross‐sectional study conducted in the general population in the United Kingdom. A web‐based survey was created using Qualtrics (Qualtrics). Participants were recruited using Prolific* (www.prolific.co), an internet platform that allows individuals to complete surveys/tasks for monetary compensation or via social media. A convenience sample of 57 vegan/vegetarian (14 male, 41 female) participants aged M = 30.67 (SD = 6.20) took part in the study. Participant sociodemographic and lifestyle characteristics are listed in Table 1. Inclusion criteria included healthy adults between ages of 18–40 years who follow a vegan or vegetarian diet. Exclusion criteria included: any history of, or taking medication for, psychiatric, sleep disorders, or neurological disorders.

**Table 1:** Baseline sociodemographic and lifestyle characteristics.

	Vegetarians (*n* = 37)	Vegans (*n* = 20)	*t/χ*2	*p* Value
Sex (F/M)	29/6	12/8	2.22	0.03
Age (years) (M ± SD)	30.70 ± 5.85	30.60 ± 6.95	0.06	0.953
BMI	25.09 ± 8.30	24.15 ± 4.65	0.06	0.646
Education (%)			6.61	0.251
GCSE/O levels	1.8	3.5		
A levels/Completed secondary	7	7		
Completed trade course/Apprenticeship	3.5	0		
Tertiary commenced (degree)	3.5	0		
Tertiary completed (degree)	22.8	17.5		
Postgraduate (Masters/PhD)	26.3	7		
Employment (%)			7.00	0.136
Employed (full‐time)	33.3	22.8		
Employed (part‐time)	24.6	5.3		
Unemployed looking for work	1.8	0		
Home duties	0	3.5		
Student	5.3	3.5		
Income (%)			7.16	0.209
<£18,000	7	5.3		
£18,000–£30,999	10.5	7		
£31,000–£51,999	21.1	10.5		
£52,000–£100,000	22.8	5.3		
>£100,000	1.8	7		
Caffeine/day (servings) (M ± SD)	2.57 ± 1.97	1.95 ± 1.40	1.22	0.226
Alcohol/day (units) (M ± SD)	2.32 ± 0.88	2.50 ± 0.95	1.07	0.293
Cigarettes/day (M ± SD)	0.11 ± 0.52	0.11 ± 0.46	0.04	0.967
Diet adherence (months) (M ± SD)	156.03 ± 117.24	46.15 ± 31.83	4.10	0.001
Physical activity/week (%)			2.17	0.704
<30 min	12.3	3.5		
30–90 min	21.1	14		
90–150 min	14	8.8		
150–300 min	12.3	3.5		
More than 300 min	5.3	5.3		

*Note*: Values reported as Mean (M) ± Standard Deviation (SD).

Abbreviations: BMI, body mass index; F, female; M, male.

### 2.2 Measures

Participants were asked to state their age, sex, weight and height, education, employment, and household income. They were also asked to report their caffeine, alcohol, and cigarette consumption, as well as dietary adherence duration and physical activity (Nordic Physical Activity Questionnaire^36^). The participants then completed questionnaires assessing personality (Big‐Five Inventory, BFI), empathy (Empathy Quotient 10, EQ‐10), mental health (Depression Anxiety Stress Scale, DASS), mental well‐being (Warwick‐Edinburgh Mental Well‐being Scales, WEMWBS), sleep (Pittsburgh Sleep Quality Index, PSQI), dietary identity (Dietarian Identity Questionnaire, DIQ), and dietary intake (EPIC Food Frequency Questionnaire [FFQ]). BMI was calculated from self‐reported height and weight BMI = weight (kg)/height (m)^2^.

#### 2.2.1 BFI

The BFI explores five personality dimensions: Extraversion, Agreeableness, Conscientiousness, Neuroticism, and Openness.^37^ The scale consists of 44 questions measuring to what degree an individual identifies with each dimension; for example, “I see myself as someone who is talkative.” The scale is measured on a five‐point Likert scale, from one (“disagree strongly”) to five (“agree strongly”), and higher scores indicate higher levels of extraversion, agreeableness, conscientiousness, neuroticism, and openness. The Cronbach *α* in the current study is 0.63.

#### 2.2.2 EQ‐10

The EQ‐10 consists of 10 questions exploring self‐reported empathy^38^; for example, “I am good at predicting how someone will feel,” and participants are required to indicate to what degree they agree with each statement. The scale is scored on a four‐point Likert scale, from one (“strongly agree”) to four (“strongly disagree”) and higher scores indicate higher levels of empathy. No Cronbach *α* was calculated in view of the brevity of this measure.

#### 2.2.3 DASS

The DASS aims to measure three dimensions: depression, anxiety, and stress.^39^ This scale consists of 42 questions, such as “I felt terrified,” requiring participants to note how frequently they identified with this statement over the past week. DASS is scored on a four‐point Likert scale, from zero (“did not apply to me at all”) to three (“applied to me very much, or most of the time”). On the depression subscale, a score of 0–9 indicates no depression, 10–13 mild depression, 14–20 moderate depression, 21–27 severe depression, and 28+ extremely severe depression. On the anxiety subscale, a score of 0–7 indicates no anxiety, 8–9 mild anxiety, 10–14 moderate anxiety, 15–19 severe anxiety, and 20+ extremely severe anxiety. On the stress subscale, a score of 0–14 indicates no stress, 15–18 mild stress, 19–25 moderate stress, 26–33 severe stress, and 34+ extremely severe stress. The Cronbach *α* in the current study is 0.98.

#### 2.2.4 WEMWBS

The WEMWBS is a 14‐item scale, exploring how psychological functioning and positive affect,^40^ and questions an individual's well‐being over the past week; for example, “I've been feeling useful.” The scale is scored on a five‐point Likert scale, ranging from one (“none of the time”) to five (“all of the time”). Total scores range from 14 to 70, with higher scores indicating better mental well‐being. The Cronbach *α* in the current study is 0.93.

#### 2.2.5 PSQI

The PSQI consists of 19 items assessing sleep quality and disturbances over the past month.^41^ The scale is split into seven components, subjective sleep quality, sleep latency, sleep duration, habitual sleep efficiency, sleep disturbances, use of sleeping medication, and daytime dysfunction; for example, “over the last month, how often have you had trouble sleeping because you feel too hot.” These components are scored individually, then summed to form a global score, determining whether a participant Is a “good sleeper” or “poor sleeper.” Higher scores indicate worse sleep quality. No Cronbach *α* was calculated in view of the brevity of this measure.

#### 2.2.6 DIQ

The DIQ consists of 34 total questions, split into nine subsections.^34^ The first subsection contains only one question regarding food groups generally not eaten. The following 33 questions are split into subsections on centrality, private regard, public regard, out‐group regard, prosocial motivation, personal motivation, moral motivation, and strictness. The scale is scored on a seven‐point Likert scale from one (“strongly disagree”) to seven (“strongly agree”), and higher scores indicate higher dietarian centrality, private regard, public regard, out‐group regard, prosocial motivation, personal motivation, moral motivation, and strictness. The Cronbach *α* in the current study is 0.95.

#### 2.2.7 EPIC FFQ

The EPIC FFQ^42^ consists of two parts; part one consists of 130 food items such as bacon and requires participants to indicate how frequently they have this food, with options ranging from “never or less than once a month” to “6+ per day.” Part two consists of questions about other foods consumed, such as brands and types of cereals, cooking fats used, meats, and added salts. FETA Software, a tool for converting FFQ data into nutrient and food group values, was also utilized in the current study.^43^

### 2.3 Procedure and ethical considerations

The survey was distributed in June 2022 using Prolific. The survey took around 30 min to complete. The current research was approved by Ethics Committee of the University of Roehampton, London, United Kingdom (PSYC/22420). All participants provided informed consent to participate in the study.

### 2.4 Statistical analyses

Statistical analyses were carried out using IBM® Statistical Package for Social Sciences (SPSS version 28; SPSS Inc.). Dietary intake, sociodemographic factors, lifestyle factors, and questionnaire measures were compared between vegetarian and vegan groups using independent sample *t*‐tests or *χ* ^2^ tests. Questionnaire measures and dietary intake are reported in Supporting Information: Tables 1S and 4S, respectively. To examine the associations between predictor variables personality traits, empathy, dietary identity and diet type, and outcome measures, mental well‐being, mental health, and sleep quality, we employed a multivariate regression approach using different models to assess the influence of specific variables on the outcome measures. First, we tested associations between predictor and outcome variables using Pearson correlations and partial correlations. Partial correlations controlling for age, sex, education, income, diet adherence duration, physical activity, BMI, and energy intake are reported in Supporting Information: Table 2S. Next, we conducted separate linear regressions to predict mental well‐being, mental health, and sleep scores from personality, empathy, dietary identity, and diet type. The models were organized into three sets: the first set predicted mental well‐being (WEMWBS: model 1, 2, and 13), the second set predicted mental health outcomes depression (DASS_D: models 3 and 4), anxiety (DASS‐A: models 5 and 6), stress (DASS‐S: models 7, 8, and 9), and the third set predicted sleep (PSQI: models 10, 11, 12, 14). To explore more complex associations between personality and dietary identity, interaction terms neuroticism × centrality was included in the regression analyses to predict stress (model 9), conscientiousness × centrality and conscientiousness × personal motivation were included to predict sleep quality (model 12). All regressions models controlled for possible confounding variables including age, sex, education, income, diet adherence duration, physical activity, BMI, and energy intake. All assumptions for the linear regression models were met. A statistical significance threshold of *p* < 0.05 (two‐tailed) was applied throughout.

## 3 RESULTS

### 3.1 Participant characteristics

Participant characteristics as a function of diet type are reported in Table 1. The groups were matched for nearly all sociodemographic and lifestyle characteristics except for sex and diet adherence duration (please see Table 1). Vegans and vegetarians did not differ in terms of BFI, EQ, DASS, WEMWBS, and PSQI scores, but they were significantly different in terms of dietary identity. While vegans reported higher levels of private regard, prosocial, personal, and moral motivation, vegetarians reported higher levels public and outgroup regard as well as strictness (please see Supporting Information: Table 1S).

### 3.2 Predicting mental and sleep health

The results from statistical models are represented in Table 2. All models significantly predicted outcome measures, except from Model 10. While higher levels of physical activity (Model 1), energy intake and dietarian private regard scores (Model 2) were significant predictors of better overall mental health (WEMWBS score), only lower levels of extraversion and higher levels of EQ predicted depression scores (Model 3). Younger age, higher levels of neuroticism, and EQ were found to be significant predictors of higher levels of anxiety (Model 5). Higher levels of neuroticism (Model 7) as well as BMI and dietarian centrality (Model 8), and neuroticism × centrality (Model 9) predicted higher levels of stress. In terms of sleep quality, higher levels of conscientiousness (Model 10), lower levels of dietarian centrality but higher levels of personal motivation and dietary strictness (Model 11), as well as conscientiousness × centrality, conscientiousness × personal motivation, and conscientiousness × strictness (Model 12) predicted lower PSQI scores (i.e., better sleep quality).

**Table 2:** Linear regression analysis predicting mental well‐being, mental health, and sleep quality scores from participant characteristics, personality, empathy, and dietary identity.

	Model		B	SE	ẞ	95% CI	*R* ^2^ (adj)
**WEMWBS**	**1**						0.289**
		(Constant)	10.745	16.940		−23.491, 44.982	
		Age (years)	−0.040	0.217	−0.027	−0.479, 0.399	
		Sex (1: M/2: F)	−0.641	2.670	−0.036	−6.037, 4.756	
		Education	−0.632	0.601	−0.140	−1.847, 0.583	
		Income	1.503	0.990	0.207	−0.498, 3.504	
		Diet adherence duration	0.017	0.011	0.215	−0.005, 0.04	
		Physical activity	2.822**	1.044	0.387	0.712, 4.932	
		BMI	−0.229	0.179	−0.186	−0.591, 0.132	
		Energy intake	0.002	0.001	0.226	−0.0004, 0.004	
		Extraversion	0.327	0.224	0.193	−0.125, 0.780	
		Agreeableness	0.497	0.281	0.259	−0.072, 1.065	
		Conscientiousness	0.051	0.261	0.034	−0.476, 0.577	
		Neuroticism	−0.206	0.194	−0.156	−0.599, 0.187	
		Openness	0.280	0.201	0.189	−0.127, 0.687	
		EQ	−0.094	0.297	−0.047	−0.694, 0.507	
	**2**						0.215*
		(Constant)	21.427	14.395		−7.715, 50.568	
		Age	−0.107	0.247	−0.074	−0.608, 0.393	
		Sex (1: M/2: F)	−0.607	2.746	−0.034	−6.166, 4.952	
		Education	−0.013	0.647	−0.003	−1.324, 1.297	
		Income	1.378	0.998	0.190	−0.644, 3.399	
		Diet adherence duration	0.018	0.015	0.218	−0.013, 0.048	
		Physical activity	1.893	1.142	0.259	−0.419, 4.205	
		BMI	−0.281	0.180	−0.227	−0.644, 0.083	
		Energy intake	0.003*	0.001	0.361	0.0004, 0.006	
		Centrality	−0.249	0.202	−0.203	−0.658, 0.160	
		Private regard	1.600**	0.627	0.636	0.331, 2.869	
		Public regard	0.097	0.303	0.049	−0.515, 0.710	
		Out‐group regard	0.130	0.159	0.157	−0.192, 0.453	
		Prosocial motivation	0.145	0.231	0.139	−0.322, 0.612	
		Personal motivation	−0.234	0.415	−0.116	−1.075, 0.606	
		Moral motivation	−0.626	0.414	−0.372	−1.465, 0.213	
		Strictness	0.326	0.307	0.171	−0.295, 0.946	
**DASS‐D**	**3**						0.500**
		(Constant)	42.141	15.212		11.397, 72.885	
		Age	−0.311	0.195	−0.201	−0.705, 0.083	
		Sex (1: M/2: F)	2.998	2.398	0.157	−1.847, 7.844	
		Education	−0.006	0.540	−0.001	−1.096, 1.085	
		Income	−0.745	0.889	−0.096	−2.542, 1.051	
		Diet adherence duration	−0.011	0.010	−0.130	−0.031, 0.009	
		Physical activity	−1.730	0.937	−0.221	−3.625, 0.164	
		BMI	0.084	0.161	0.064	−0.240, 0.409	
		Energy intake	0.002	0.001	0.214	−0.0001, 0.004	
		Extraversion	−0.510**	0.201	−0.280	−0.916, −0.103	
		Agreeableness	−0.497	0.252	−0.243	−1.007, 0.013	
		Conscientiousness	−0.237	0.234	−0.149	−0.709, 0.236	
		Neuroticism	0.276	0.175	0.195	−0.077, 0.629	
		Openness	−0.146	0.181	−0.092	−0.512, 0.219	
		EQ	0.696**	0.267	0.328	0.157, 1.235	
	**4**						0.308**
		(Constant)	22.439	14.466		−6.847, 51.724	
		Age	−0.240	0.248	−0.155	−0.743, 0.263	
		Sex (1: M/2: F)	2.786	2.760	0.145	−2.801, 8.373	
		Education	−0.279	0.650	−0.058	−1.596, 1.038	
		Income	−1.049	1.003	−0.135	−3.080, 0.982	
		Diet adherence duration	−0.013	0.015	−0.148	−0.043, 0.018	
		Physical activity	−1.094	1.148	−0.140	−3.418, 1.229	
		BMI	0.217	0.180	0.164	−0.149, 0.582	
		Energy intake	0.001	0.001	0.131	−0.002, 0.004	
		Centrality	0.349	0.203	0.266	−0.062, 0.760	
		Private regard	−1.247	0.630	−0.463	−2.522, 0.028	
		Public regard	−0.514	0.304	−0.241	−1.130, 0.102	
		Out‐group regard	0.094	0.160	0.106	−0.230, 0.418	
		Prosocial motivation	0.011	0.232	0.010	−0.458, 0.480	
		Personal motivation	−0.078	0.417	−0.036	−0.922, 0.767	
		Moral motivation	0.229	0.417	0.127	−0.614, 1.073	
		Strictness	−0.084	0.308	−0.041	−0.708, 0.540	
**DASS‐A**	**5**						0.516**
		(Constant)	10.912	11.273		−11.872, 33.696	
		Age	−0.321*	0.145	−0.276	−0.613, −0.029	
		Sex (1: M/2: F)	1.899	1.777	0.132	−1.692, 5.490	
		Education	−0.079	0.400	−0.022	−0.888, 0.729	
		Income	−0.312	0.659	−0.053	−1.644, 1.019	
		Diet adherence duration	−0.005	0.007	−0.078	−0.020, 0.010	
		Physical activity	−0.587	0.695	−0.100	−1.992, 0.817	
		BMI	0.033	0.119	0.033	−0.208, 0.274	
		Energy intake	0.001	0.001	0.192	−0.0002, 0.003	
		Extraversion	−0.182	0.149	−0.133	−0.483, 0.119	
		Agreeableness	−0.118	0.187	−0.077	−0.496, 0.260	
		Conscientiousness	−0.165	0.173	−0.138	−0.516, 0.185	
		Neuroticism	0.407**	0.129	0.382	0.146, 0.669	
		Openness	0.016	0.134	0.013	−0.255, 0.287	
		EQ	0.468*	0.198	0.293	0.068, 0.868	
	**6**						0.333**
		(Constant)	7.764	10.702	0	−13.901, 29.429	
		Age	−0.316	0.184	−0.272	−0.688, 0.056	
		Sex (1: M/2: F)	3.046	2.042	0.211	−1.086, 7.179	
		Education	−0.119	0.481	−0.033	−1.093, 0.855	
		Income	−0.438	0.742	−0.075	−1.941, 1.065	
		Diet adherence duration	−0.007	0.011	−0.111	−0.030, 0.015	
		Physical activity	−0.585	0.849	−0.099	−2.304, 1.134	
		BMI	0.158	0.134	0.159	−0.112, 0.429	
		Energy intake	0.001	0.001	0.205	−0.001, 0.004	
		Centrality	0.276	0.150	0.279	−0.028, 0.580	
		Private regard	−0.773	0.466	−0.381	−1.716, 0.171	
		Public regard	−0.241	0.225	−0.150	−0.696, 0.215	
		Out‐group regard	0.156	0.118	0.233	−0.083, 0.396	
		Prosocial motivation	−0.021	0.171	−0.025	−0.368, 0.326	
		Personal motivation	0.021	0.309	0.013	−0.603, 0.646	
		Moral motivation	0.363	0.308	0.267	−0.261, 0.986	
		Strictness	−0.149	0.228	−0.097	−0.611, 0.313	
**DASS‐S**	**7**						0.577**
		(Constant)	−0.478	13.984		−28.740, 27.784	
		Age	−0.231	0.179	−0.149	−0.593, 0.132	
		Sex (1: M/2: F)	0.275	2.204	0.014	−4.179, 4.730	
		Education	0.006	0.496	0.001	−0.997, 1.009	
		Income	0.321	0.817	0.041	−1.331, 1.973	
		Diet adherence duration	−0.001	0.009	−0.014	−0.020, 0.017	
		Physical activity	−1.055	0.862	−0.135	−2.797, 0.687	
		BMI	0.153	0.148	0.116	−0.145, 0.452	
		Energy intake	0.001	0.001	0.142	−0.001, 0.003	
		Extraversion	−0.082	0.185	−0.045	−0.456, 0.291	
		Agreeableness	−0.215	0.232	−0.105	−0.684, 0.254	
		Conscientiousness	−0.169	0.215	−0.107	−0.604, 0.266	
		Neuroticism	0.800**	0.161	0.565	0.476, 1.124	
		Openness	0.091	0.166	0.057	−0.245, 0.427	
		EQ	0.480	0.245	0.227	−0.016, 0.976	
	**8**						0.298**
		(Constant)	0.445	14.568		−29.047, 29.936	
		Age	−0.388	0.250	−0.251	−0.894, 0.119	
		Sex (1: M/2: F)	1.918	2.779	0.100	−3.708, 7.544	
		Education	0.335	0.655	0.070	−0.991, 1.661	
		Income	−0.080	1.010	−0.010	−2.126, 1.965	
		Diet adherence duration	−0.010	0.015	−0.121	−0.041, 0.020	
		Physical activity	−1.234	1.156	−0.158	−3.574, 1.106	
		BMI	0.375*	0.182	0.283	0.007, 0.742	
		Energy intake	0.002	0.001	0.184	−0.001, 0.005	
		Centrality	0.483*	0.204	0.368	0.069, 0.897	
		Private regard	−0.979	0.634	−0.364	−2.263, 0.305	
		Public regard	−0.330	0.306	−0.155	−0.950, 0.291	
		Out‐group regard	0.197	0.161	0.222	−0.129, 0.524	
		Prosocial motivation	−0.062	0.233	−0.055	−0.534, 0.411	
		Personal motivation	0.313	0.420	0.145	−0.537, 1.164	
		Moral motivation	0.275	0.419	0.152	−0.574, 1.124	
		Strictness	0.110	0.310	0.054	−0.519, 0.738	
	**9**						0.489**
		(Constant)	1.206	8.14		−15.189, 17.601	
		Age	−0.410*	0.181	−0.265	−0.776, −0.045	
		Sex (1: M/2: F)	2.064	2.095	0.108	−2.155, 6.283	
		Education	0.564	0.514	0.117	−0.472, 1.600	
		Income	−0.438	0.832	−0.056	−2.113, 1.237	
		Diet adherence duration	−0.001	0.010	−0.008	−0.020, 0.019	
		Physical activity	−1.692	0.867	−0.217	−3.438, 0.054	
		BMI	0.311*	0.135	0.235	0.039, 0.583	
		Energy intake	0.002	0.001	0.222	0.00009, 0.004	
		Neuroticism × Centrality	0.017**	0.004	0.477	0.010, 0.024	
**PSQI**	**10**						0.131
		(Constant)	18.034	5.196		7.539, 28.528	
		Age	0.034	0.067	0.084	−0.101, 0.170	
		Sex (1: M/2: F)	1.274	0.823	0.252	−0.388, 2.935	
		Education	0.092	0.187	0.072	−0.286, 0.470	
		Income	0.154	0.304	0.075	−0.460, 0.768	
		Diet adherence duration	0.001	0.003	0.025	−0.006, 0.007	
		Physical activity	−0.439	0.324	−0.213	−1.094, 0.216	
		BMI	−0.030	0.055	−0.087	−0.142, 0.082	
		Energy intake	0.001	0.0003	0.198	−0.0002, 0.001	
		Extraversion	−0.071	0.067	−0.147	−0.205, 0.064	
		Agreeableness	−0.016	0.081	−0.030	−0.180, 0.147	
		Conscientiousness	−0.230**	0.079	−0.549	−0.389, −0.07	
		Neuroticism	−0.007	0.060	−0.020	−0.130, 0.115	
		Openness	−0.049	0.061	−0.117	−0.173, 0.075	
	**11**						0.230*
		(Constant)	13.840	4.025		5.692, 21.988	
		Age	0.033	0.069	0.080	−0.107, 0.173	
		Sex (1: M/2: F)	1.576*	0.768	0.312	0.021, 3.130	
		Education	−0.036	0.181	−0.028	−0.402, 0.331	
		Income	−0.335	0.279	−0.164	−0.900, 0.230	
		Diet adherence duration	0.004	0.004	0.170	−0.005, 0.012	
		Physical activity	−0.357	0.319	−0.173	−1.004, 0.289	
		BMI	−0.003	0.050	−0.008	−0.104, 0.099	
		Energy intake	0.0004	0.0004	0.170	−0.0004, 0.001	
		Centrality	0.167**	0.056	0.483	0.053, 0.281	
		Private regard	−0.099	0.175	−0.139	−0.453, 0.256	
		Public regard	−0.136	0.085	−0.241	−0.307, 0.036	
		Out‐group regard	−0.039	0.045	−0.167	−0.129, 0.051	
		Prosocial motivation	0.029	0.064	0.098	−0.101, 0.160	
		Personal motivation	−0.319**	0.116	−0.561	−0.554, −0.084	
		Moral motivation	0.080	0.116	0.169	−0.154, 0.315	
		Strictness	−0.234**	0.086	−0.435	−0.408, −0.060	
	**12**						0.306**
		(Constant)	10.475	2.529		5.375, 15.575	
		Age	0.049	0.062	0.119	−0.078, 0.175	
		Sex (1: M/2: F)	1.425*	0.686	0.282	0.041, 2.81	
		Education	−0.003	0.158	−0.002	−0.321, 0.315	
		Income	−0.034	0.267	−0.017	−0.572, 0.504	
		Diet adherence duration	0.001	0.003	0.029	−0.006, 0.007	
		Physical activity	−0.628*	0.281	−0.305	−1.196, −0.061	
		BMI	−0.017	0.048	−0.048	−0.113, 0.079	
		*Energy intake*	*0.001*	0.0003	0.220	−0.00006, 0.001	
		*Conscientiousness* × *Cent*	*0.005* **	0.001	0.59	0.002, 0.008	
		Conscientiousness × Pers motiv	−0.008**	0.002	−0.583	−0.013, −0.003	

Abbreviations: DASS, Depression (DASS‐D), Anxiety (DASS‐A) and Stress (DASS‐S) Scores; EQ, empathy quotient; F, female; M, male; PSQI, Pittsburgh Sleep Quality Index; WEMWBS, Warwick‐Edinburgh Mental Well‐being Scales.

* *p* < 0.05

** *p* < 0.001.

As private regard, personal motivation and strictness scores were (i) significantly different in vegans compared to vegetarians and (ii) significant predictors of WEMWBS and PSQI, we also added diet type as additional covariate to linear regression models 13 and 14, however, diet type was not found to predict WEMWBS and PSQI (Please see Supporting Information: Table 3S).

## 4 DISCUSSION

The aim of this preliminary study was to examine the associations between diet type, dietary identity, personality, and mental and sleep health in a sample of vegetarian and vegan participants. Overall, we found that personality traits and components of dietary identity significantly predicted both sleep and mental health. Furthermore, our results showed that complex interactions between dietary identity and personality traits predicted higher stress and worse sleep quality.

### 4.1 Personality traits and mental outcomes

Our analysis showed that personality traits did not predict overall mental well‐being (WEMWBS scores) (Model 1), however, neuroticism, empathy, and extraversion traits predicted specific mental health outcomes. Precisely, lower levels of extraversion and higher levels of empathy predicted depression (Model 3), higher levels of neuroticism and empathy predicted anxiety (Model 5), and higher levels of neuroticism predicted stress (Model 7).

We found that higher levels of depression and anxiety were predicted by higher levels of empathy. Research studies have found that vegans and vegetarians tend to report higher levels of empathy compared to omnivores^14^ which may relate to higher levels of empathic concern for the welfare of humans and animals reported for individuals who follow a plant‐based dietary pattern.^44^, ^45^, ^46^ However, it is well known that maladaptive components of empathy (such as personal distress, a maladaptive form affective empathy and interpersonal guilt, a maladaptive form of cognitive empathy^47^) may confer risk for anxiety and depression.^25^, ^26^, ^47^ This is even more pronounced for affective empathy as evidence from meta‐analyses show that higher levels of affective empathy (an emotional response that allows an individual to perceive and experience another's emotional state), but not cognitive empathy (engagement with higher cognitive processes that allows an individual to understand the emotions of others), is associated with depression.^25^ Therefore, future research should include analyses of both cognitive and affective components empathy to better understand the risk for depression in vegans and vegetarian populations.

We found that lower levels of extraversion predicted higher depression scores, in line with results reported by Hakulinen et al.^48^ Yet in the research literature, associations between extraversion and diet types are not consistent (i.e., some studies report higher levels of extraversion in vegetarians and vegans compared to omnivores,^16^, ^49^, ^50^ others report no differences^51^ or higher levels of extraversion were found to be associated with meat consumption.^12^, ^13^ Additionally, research to date has shown positive associations between introversion and depression in unselected samples.^52^, ^53^ Hence, it is crucial to understand extraversion/introversion traits in relation to diet type to better understand our findings. Regardless, it is important to note that inconsistencies may be described by lower levels of the personality trait hierarchy. Domain‐level traits (neuroticism, consciousness, agreeableness, extraversion, openness) predict a wider range of phenomena at a modest level, while aspect and facet‐level traits (affiliation, positive affectivity, energy, and ascendence^54^) predict a narrower range of phenomena, but with a higher degree of accuracy and strength.^55^, ^56^ Converging evidence comes from Tan et al.^16^ showing that vegetarian and vegans report higher levels of energy, a facet‐level trait within the extraversion domain, which may explain higher scores for extraversion overall reported for this population. Additionally, specific associations of psychopathology may exist at the lower level of the personality trait hierarchy.^57^ For instance, depression is found to be associated with lack of positive affectivity^58^ and low levels of positive emotionality are found to prospectively predict depression.^59^ Although we cannot infer causality between low extraversion and higher levels of depression in our sample, one study found that vegetarians reported lower self‐esteem, lower psychological adjustment, and more negative moods compared to omnivores.^60^ This indicates that there may be associations between depression and aspect and facet‐level personality traits within the extraversion domain that are specific to vegan and vegetarian populations.

For depression, we expected that neuroticism would predict higher depression scores in our sample population given that neuroticism is strongly associated with mental health disorders and risk of prospective diagnosis^19^, ^61^, ^62^ and the association between neuroticism and depression has been reported in vegan and vegetarian populations.^17^ However, our study may have been underpowered to test this association. Further, the evidence on the effect of vegetarian and vegan diets on depression is conflicting, with some studies showing a higher risk of depression in vegans and vegetarians,^7^, ^9^ while others showing no association^63^ or that a vegetarian/vegan diet may be beneficial for depression outcomes.^8^ The inconsistency in study outcomes may be due, in part, to the large heterogeneity observed in the studies analyzed. Notably variation in the classification of diet types, sampling population and small effect sizes may have contributed to outcomes.^7^, ^8^, ^10^ Instead, we observed that neuroticism predicted only anxiety and stress outcomes. As shown in previous research in clinical populations,^64^, ^65^ it may be the case that there is a specific pathway (such as worry and/or shame) linking neuroticism to anxiety in vegans and vegetarians.

For anxiety, higher levels were predicted by higher levels of neuroticism, empathy, and younger age. We also expected that there may be an interaction between empathy and neuroticism in predicting anxiety, given the association between empathy and internalizing disorders discussed above. Despite the lack of association, the interaction between empathetic and personality traits warrants further investigation, specifically as it is not clear whether these traits precede the selection of a vegan and vegetarian diet or whether following the diet predisposes an individual to risk of anxiety and depression disorders. An important finding was that our model did account for the effects of age, as younger vegans and vegetarians may be an increased risk of anxiety‐related disorders.^7^

In terms of overall mental well‐being (WEMWBS scores), our analysis showed that personality traits did not predict better mental well‐being outcome. Yet traits, such as conscientiousness and extraversion, are considered protective factors and offer a positive influence on mental health.^20^, ^21^ It is important to note that the WEMWBS is a measure of mental well‐being focusing entirely on positive aspects of mental health,^40^ whereas the DASS do not only measure separate domains of mental health, but also focus on negative aspects of mental health.^39^ Hence, psychometric, as well as conceptual^66^, ^67^, ^68^ (mental well‐being vs. mental ill health) differences between these two measures might contribute to this inconsistency in our results.

### 4.2 Personality traits and sleep health

Consistent with previous research,^27^, ^28^, ^29^, ^30^ we found that higher levels of conscientiousness (Model 10) predicted lower PSQI scores (i.e., better sleep quality). This result could be partially attributable to better mental and physical health outcomes^69^ and stress reactivity,^70^ reported by conscientious individuals. On the other hand, unlike previous research,^29^, ^30^ we did not observe associations between sleep quality and neuroticism. However, it is important to note that, these studies did not take diet type into consideration. Additionally, it is unclear whether neuroticism (i) is a direct predictor of sleep, and/or (ii) it moderates associations between related psychological processes and sleep, as after accounting for daily rumination and negative affect, the association between neuroticism and sleep has shown to be diminished.^71^ Future studies examining personality and sleep should consider controlling for rumination, affect, and/or mental health‐related variables.

### 4.3 Dietarian identity and mental and sleep health outcomes

In terms of dietarian identity, we found that higher private regard predicted better overall mental health (Model 2), but not depression, anxiety, and stress symptoms (Models 4, 6, and 8). As omnivores have been shown to have fewer positive feelings toward other omnivores than plant‐based eaters have for other plant‐based eaters,^33^ this positivity toward other plant‐based eaters may be a protective factor for overall mental health and well‐being. For instance, it has been shown that having shared identities^72^ and group positive affect (i.e., positive affect among group members)^73^ are associated with resilience and positive emotions. Moreover, although overall mental well‐being and mental health are highly correlated,^66^ the reason why we did not observe associations between dietarian identity and depression and anxiety, needs further exploration in a more representative sample.

We also showed that higher levels of stress, however, was predicted by higher levels of dietarian centrality (Model 8) and neuroticism and centrality interaction predicted higher levels of stress (Model 9). Given its socially nonnormative status, plant‐based identity (compared to omnivorous identity) may be more salient and more central to one's self, therefore, this nonnormative identity (along with neuroticism) may have contributed to higher levels of stress experienced by plant‐based eaters.^74^, ^75^

Regarding sleep quality, lower levels of dietarian centrality, but higher levels of personal motivation and dietary strictness (Model 11), and interactions between conscientiousness and (i) centrality, (ii) personal motivation, and (iii) strictness (Model 12) predicted lower PSQI scores (i.e., better sleep quality). As there is a bidirectional relationship between mental health and poor sleep outcomes,^76^, ^77^, ^78^, ^79^ our findings showing associations between higher levels of dietarian centrality and higher levels of stress in our study could be explained by this bidirectional relationship. Our findings showing associations between higher levels of personal motivation and strictness (along with higher levels of conscientiousness) and better sleep quality we believe, could be explained by positive lifestyle choices (e.g., healthy eating, physical exercise, not smoking, etc.) made by conscientiousness individuals who strictly adhere to plant‐based diets for reasons related to health and well‐being. For instance, compared to vegans following the diet for ethical reasons, those doing so for health reasons reported eating more fruit and fewer sweets,^80^ which are known to be associated with better sleep outcomes.^81^, ^82^ Additionally, it is possible that these individuals would be involved in better sleep hygiene practices as evidenced by Duggan et al.^29^

### 4.4 Limitations

The current study has a number of limitations which need to be considered when interpreting the findings. First, due to the cross‐sectional nature of the study, we could not determine causal relationships, hence, reverse causation is also possible. Second, in the current study, nutrient intakes were measured by using a tool that processes dietary data from the FFQ. As dietary intake measures rely on the ability of participants to recall and report, under­reporting may be possible.^83^ Third, this study is limited to the British population, and it has been shown that diet type and mental health associations might not be associated the same way across cultures.^84^ Fourth, although the effect sizes of some of the associations are small, small effects may nevertheless be socially important.^85^ Fifth, it should be noted that the Cronbach's *α* for the BFI was rather low, however, similarly low values for the BFI were reported before^12^, ^13^, ^86^ and this could be explained by the general weakness of the BFI and its brevity. Finally, as the current study is preliminary and skewed toward females, our findings should be replicated in bigger sex‐matched samples. Therefore, future cross‐cultural longitudinal studies in bigger cohorts, preferably by using objective measures (e.g., biomarkers) to assess dietary intake, as well as mental and sleep health are warranted to uncover the complex associations between diet type, personality, dietary identity, and mental and sleep health outcomes.

## 5 CONCLUSION

The current preliminary study extended our knowledge regarding the predictors of mental and sleep health in plant‐based eaters and highlighted the importance of considering dietarian identity traits, in addition to personality traits, in predicting health outcomes.

## AUTHOR CONTRIBUTIONS

**Christle Coxon**: Conceptualization; data curation; formal analysis; investigation; methodology; project administration; supervision; writing—original draft; writing—review and editing. **Piril Hepsomali**: Conceptualization; data curation; formal analysis; investigation; methodology; project administration; supervision; writing—original draft; writing—review and editing. **Karen Brandt**: Conceptualization; writing—original draft; writing—review and editing. **David Vauzour**: Writing—review and editing. **Adele Costabile**: Writing—review and editing.

## CONFLICT OF INTEREST STATEMENT

The authors declare no conflict of interest.

## TRANSPARENCY STATEMENT

The lead author Christle Coxon affirms that this manuscript is an honest, accurate, and transparent account of the study being reported; that no important aspects of the study have been omitted; and that any discrepancies from the study as planned (and, if relevant, registered) have been explained.

## Supporting information

Supporting information.

## Data Availability

The authors confirm that the data supporting the findings of this study are available on request.

## ENDNOTE

* Respondents completing surveys through Prolific (i) were found to be more naïve, diverse, less dishonest compared to participants in other platforms and (ii) produced high‐quality data.^35^
