# Supplementary material for: Personality, dietary identity, mental and sleep health in vegans and vegetarians: A preliminary cross‐sectional study
Source: Health Sci Rep. 2023 Aug 22;6(8):e1525. doi: 10.1002/hsr2.1525 (PMC10444972; doi:10.1002/hsr2.1525)
Supplement: Supplementary file 1 — Supporting information. [file HSR2-6-e1525-s001.docx]

# Supplementary Materials

### Table 1S: Comparison of scores for mental wellbeing, personality traits, empathy, mental health, and dietarian identity in vegetarian and vegans

|  | **Vegetarians (n=37)** | | **Vegans (n=20)** | |  |  |  |  |
| --- | --- | --- | --- | --- | --- | --- | --- | --- |
|  | ***M*** | ***SD*** | ***M*** | ***SD*** | ***t*** | ***df*** | ***p*** | ***Cohen’s d*** |
| ***BFI*** |  |  |  |  |  |  |  |  |
| Extraversion | 23.595 | 5.341 | 21.950 | 4.925 | 1.139 | 55 | 0.260 | 0.316 |
| Agreeableness | 34.135 | 4.756 | 32.700 | 5.162 | 1.055 | 55 | 0.296 | 0.293 |
| Conscientiousness | 33.243 | 6.171 | 31.750 | 5.857 | 0.887 | 55 | 0.379 | 0.246 |
| Neuroticism | 25.270 | 6.665 | 25.750 | 6.973 | -0.255 | 55 | 0.800 | -0.071 |
| Openness | 35.568 | 6.735 | 37.950 | 5.155 | -1.377 | 55 | 0.174 | -0.382 |
| ***EQ*** | 10.730 | 4.501 | 10.150 | 4.998 | 0.446 | 55 | 0.657 | 0.124 |
| ***DASS*** |  |  |  |  |  |  |  |  |
| Depression | 6.730 | 9.500 | 9.500 | 9.534 | -1.049 | 55 | 0.299 | -0.291 |
| Anxiety | 5.730 | 7.089 | 7.200 | 7.452 | -0.734 | 55 | 0.466 | -0.204 |
| Stress | 11.216 | 11.098 | 11.750 | 8.097 | -0.189 | 55 | 0.851 | -0.053 |
| ***DIQ*** |  |  |  |  |  |  |  |  |
| Centrality | 23.595 | 6.805 | 26.850 | 7.903 | -1.628 | 55 | 0.109 | -0.452 |
| Private regard | 14.081 | 3.585 | 16.250 | 3.275 | -2.245 | 55 | 0.029 | -0.623 |
| Public regard | 11.027 | 4.375 | 7.100 | 3.553 | 3.443 | 55 | 0.001 | 0.956 |
| Out-Group regard | 36.541 | 9.783 | 30.150 | 13.084 | 2.087 | 55 | 0.042 | 0.579 |
| Prosocial motivation | 28.486 | 9.239 | 33.850 | 6.293 | -2.317 | 55 | 0.024 | -0.643 |
| Personal motivation | 14.405 | 4.752 | 17.300 | 3.230 | -2.432 | 55 | 0.018 | -0.675 |
| Moral motivation | 13.216 | 5.324 | 16.600 | 4.512 | -2.410 | 55 | 0.019 | -0.669 |
| Strictness | 17.865 | 3.772 | 15.150 | 6.434 | 2.013 | 55 | 0.049 | 0.559 |
| ***PSQI*** | 9.378 | 2.178 | 9.850 | 2.996 | -0.682 | 55 | 0.498 | -0.189 |
| ***WEMWBS*** | 47.351 | 9.598 | 46.400 | 8.488 | 0.371 | 55 | 0.712 | 0.103 |

Abbreviations: WEMWBS: Warwick-Edinburgh Mental Wellbeing Scales; DASS: Depression (DASS-D), Anxiety (DASS-A) and Stress (DASS-S) Scores; EQ: Empathy Quotient; PSQI: Pittsburgh Sleep Quality Index.

### Table 2S: Partial correlations between personality, dietary identity, mental, and sleep health variables

|  | **WEMWBS** | **DASS-D** | **DASS-A** | **DASS-S** | **PSQI** |
| --- | --- | --- | --- | --- | --- |
| ***BFI*** |  |  |  |  |  |
| Extraversion | 0.279* | -0.299* | -0.166 | -0.133 | -0.143 |
| Agreeableness | 0.37 | -0.31* | -0.16 | -0.252 | -0.203 |
| Conscientiousness | 0.149 | -0.333* | -0.341** | -0.365** | -0.435** |
| Neuroticism | -0.345** | 0.379** | 0.478** | 0.642** | 0.183 |
| Openness | 0.263 | -0.075 | 0.028 | 0.012 | -0.069 |
| ***EQ*** | 0.219 | 0.105 | 0.187 | 0.087 | -0.029 |
| ***DIQ*** |  |  |  |  |  |
| Centrality | -0.142 | 0.128 | 0.182 | 0.309* | 0.321* |
| Private regard | 0.13 | -0.201 | -0.121 | -0.025 | 0.057 |
| Public regard | -0.049 | -0.159 | -0.091 | -0.171 | -0.121 |
| Out-Group regard | 0.145 | 0.134 | 0.15 | 0.094 | -0.198 |
| Prosocial motivation | 0.041 | -0.055 | -0.008 | 0.08 | -0.032 |
| Personal motivation | -0.082 | -0.056 | 0.051 | 0.159 | -0.126 |
| Moral motivation | -0.143 | -0.088 | 0.011 | 0.049 | 0.129 |
| Strictness | 0.185 | -0.012 | -0.097 | 0.064 | -0.189 |

Controlled for: age, sex, education, income, diet adherence duration, physical activity, BMI, and energy intake. Abbreviations:BFI: Big Five Inventory; EQ: Empathy Quotient; DIQ: Dietarian Identity Questionnaire.

*Correlation is significant at the 0.05 level (2-tailed).

**Correlation is significant at the 0.01 level (2-tailed).

### Table 3S: Linear regression analysis predicting mental wellbeing and sleep quality by participant characteristics and dietarian identity and diet type.

|  | **Model** |  | **B** | **SE** | **ẞ** | **95%CI** | ***R*^2^ (adj)** |
| --- | --- | --- | --- | --- | --- | --- | --- |
| **WEMWBS** | **13** |  |  |  |  |  | .194 |
|  |  | (Constant) | 22.271 | 17.422 |  | -13.03, 57.572 |  |
|  |  | Age (years) | -0.104 | 0.254 | -0.072 | -0.618, 0.41 |  |
|  |  | Sex (1:M/2: F) | -0.619 | 2.786 | -0.035 | -6.263, 5.026 |  |
|  |  | Education | -0.017 | 0.657 | -0.004 | -1.348, 1.314 |  |
|  |  | Income | 1.373 | 1.013 | 0.189 | -0.68, 3.426 |  |
|  |  | Diet adherence duration | 0.017 | 0.016 | 0.212 | -0.015, 0.049 |  |
|  |  | Physical activity | 1.894 | 1.158 | 0.260 | -0.451, 4.24 |  |
|  |  | BMI | -0.281 | 0.182 | -0.227 | -0.65, 0.088 |  |
|  |  | Energy intake | 0.003* | 0.001 | 0.361 | 0.004, 0.006 |  |
|  |  | Centrality | -0.247 | 0.205 | -0.202 | -0.663, 0.168 |  |
|  |  | Private regard | 1.593* | 0.641 | 0.634 | 0.295, 2.891 |  |
|  |  | Public regard | 0.087 | 0.328 | 0.044 | -0.577, 0.752 |  |
|  |  | Out-Group regard | 0.130 | 0.161 | 0.157 | -0.197, 0.457 |  |
|  |  | Prosocial motivation | 0.145 | 0.234 | 0.139 | -0.328, 0.618 |  |
|  |  | Personal motivation | -0.230 | 0.424 | -0.114 | -1.088, 0.629 |  |
|  |  | Moral motivation | -0.625 | 0.420 | -0.371 | -1.476, 0.227 |  |
|  |  | Strictness | 0.320 | 0.318 | 0.168 | -0.324, 0.964 |  |
|  |  | Diet type | -0.270 | 3.049 | -0.015 | -6.447, 5.907 |  |
| **PSQI** | **14** |  |  |  |  |  | .225* |
|  |  | (Constant) | 11.548 | 4.823 |  | 1.776, 21.320 |  |
|  |  | Age | 0.023 | 0.070 | 0.057 | -0.119, 0.165 |  |
|  |  | Sex (1:M/2: F) | 1.607* | 0.771 | 0.318 | 0.044, 3.169 |  |
|  |  | Education | -0.026 | 0.182 | -0.021 | -0.395, 0.342 |  |
|  |  | Income | -0.322 | 0.280 | -0.157 | -0.890, 0.246 |  |
|  |  | Diet adherence duration | 0.005 | 0.004 | 0.226 | -0.004, 0.014 |  |
|  |  | Physical activity | -0.362 | 0.320 | -0.176 | -1.011, 0.287 |  |
|  |  | BMI | -0.002 | 0.050 | -0.006 | -0.104, 0.100 |  |
|  |  | Energy intake | 0.000 | 0.000 | 0.176 | -0.0004, 0.001 |  |
|  |  | Centrality | 0.163* | 0.057 | 0.473 | 0.048, 0.279 |  |
|  |  | Private regard | -0.079 | 0.177 | -0.111 | -0.438, 0.280 |  |
|  |  | Public regard | -0.108 | 0.091 | -0.192 | -0.292, 0.076 |  |
|  |  | Out-Group regard | -0.038 | 0.045 | -0.163 | -0.129, 0.052 |  |
|  |  | Prosocial motivation | 0.029 | 0.065 | 0.098 | -0.102, 0.160 |  |
|  |  | Personal motivation | -0.331* | 0.117 | -0.583 | -0.569, -0.094 |  |
|  |  | Moral motivation | 0.076 | 0.116 | 0.160 | -0.160, 0.312 |  |
|  |  | Strictness | -0.218* | 0.088 | -0.406 | -0.396, -0.040 |  |
|  |  | Diet type | 0.733 | 0.844 | 0.140 | -0.977, 2.443 |  |

**p < 0.001, *p < 0.05: Abbreviations: WEMWBS: Warwick-Edinburgh Mental Wellbeing Scales; DASS: Depression (DASS-D), Anxiety (DASS-A) and Stress (DASS-S) Scores; EQ: Empathy Quotient; PSQI: Pittsburgh Sleep Quality Index; F: female; M: Male

# Supplementary Introduction: Nutrition status, diet type, mental and sleep health

Nutritional intake and status may play an important role in mental and sleep health. In the general population, low diet quality, irrespective of diet type, is linked with poor mental health and sleep health outcomes (Hepsomali & Groeger, 2021). Vegan and vegetarian diets are of a higher nutritional quality and found to be lower in total energy, lower in saturated fat and cholesterol, and higher in carbohydrate and fibre (Allès et al., 2017; Clarys et al., 2014; Hargreaves et al., 2020). In terms of nutritional intake, a recent systematic review found that compared to meat-eaters, vegetarians and vegans had a higher intake of dietary fibre, polyunsaturated fatty acids, Vitamin C, E, and magnesium. However, vegetarians and vegans were found to have lower intakes of protein, Vitamin B12, D, iron, zinc, iodine, calcium, eicosapentaenoic acid (EPA) and docosahexaenoic acid (DHA) (Neufingerl & Eilander, 2021). Similarly, studies assessing nutritional status indicate that compared to meat-eaters, vegetarians and vegans were found to have lower levels of Vitamin B12, D, iodine (Larsson & Johansson, 2002; Neufingerl & Eilander, 2021; Weikert et al., 2020), and calcium, copper, and zinc (Gajski et al., 2018). Nutrient deficiency was reported for Vitamin B6, B3, and zinc in vegans and vegetarians (Foster et al., 2013; Schüpbach et al., 2015). One study reported that in vegetarians and vegans, Vitamin B12 deficiency was low, due in part to supplementation, yet across the population, functional markers (methylmalonic acid (MMA) and homocysteine (Hcy)) indicated subclinical deficiency in over a third of the sample population (Gallego-Narbón et al., 2019). Moreover, vegans and vegetarians were found to have a higher prevalence of estimated nutritional inadequacy in Vitamin B12, D, calcium, iodine, iron (in women) and zinc, long-chain omega-3 fatty acids eicosapentaenoic acid (EPA) and docosahexaenoic acid (DHA) (Neufingerl & Eilander, 2021).

Vegan diets are more restrictive and exclude all forms of animal products, therefore, individuals who consume vegan diets may be at higher risk of nutritional deficiencies (Sakkas et al., 2020). A systematic review found that compared to omnivores and vegetarians, vegans are found to have the lower intakes of Vitamins B2, Niacin (B3), B12, D, iodine, zinc, calcium, potassium, selenium. Moreover, Vitamin B12 and calcium intakes were below nutritional recommendations (Dimitra et al., 2021).While another study found that serum Vitamin B12 levels were lowest, while folate were highest, in vegan participants (Gilsing et al., 2010). The concern is that suboptimal nutrient status (in both vegans and vegetarians) may be associated with risk of poor neuropsychiatric outcomes (Kapoor et al., 2017).

Although it is clear that different diet types are associated with different nutrient intake profiles, these differences in dietary intake did not consistently affect mental and sleep health outcomes. Systematic and meta-analytic evidence showed that adherence to a vegetarian or vegan diets was associated with increased risk of depression (Iguacel et al., 2021), but with lower anxiety scores (Iguacel et al., 2021). However, a systematic review by Jain et al. (2022), has also shown beneficial effects of vegetarian and vegan diets on depression. Converging experimental evidence also supports these findings by showing that restricting meat, fish, and poultry for two weeks improved stress symptoms in omnivores (Beezhold & Johnston, 2012). Similarly, adherence to plant-forward diets (e.g., the Mediterranean diet) was shown to be associated with better mental health outcomes (Firth et al., 2019; Hepsomali & Groeger, 2021; Jacka et al., 2010). A similar pattern exists for sleep outcomes. Some studies reported null findings (Sengul, 2022), whereas others reported that higher percentage of plant-based protein intake and adherence to plant-forward diets were associated with better sleep quality (Crawford et al., 2017; Godos et al., 2019; Hepsomali & Groeger, 2021). The conflicting results observed in the studies summarised above might be explained due to the differences in research design, sampling technique, measures used, and covariates included in the statistical models.

# Supplementary analysis and results:

Vegetarian and vegan groups were compared on dietary intake using independent sample t-tests. Dietary intake as a function of diet type is reported in supplementary table 3.

Vegans consume less lactose and Vit B12 compared to vegetarians. On the other hand, vegetarians consume less galactose, polyunsaturated fatty acid (PUFA) total, Vitamin B1, Vitamin E, copper, magnesium, and manganese compared to vegans. For all other nutrients *p*’s>0.1.

## Table 4S. Comparison of dietary intake in Vegans and Vegetarians.

|  | **Vegetarians (n=37)** | | | **Vegans (n=20)** | | |  |  |  |  |
| --- | --- | --- | --- | --- | --- | --- | --- | --- | --- | --- |
|  | ***M*** | ***SD*** | ***M*** | | ***SD*** | ***t*** | | ***df*** | ***p*** | ***Cohen’s d*** |
| ***Energy Intake*** *(kcal)* | 1485.786 | 675.307 | 1712.796 | | 1379.933 | -0.836 | | 55 | 0.407 | -0.232 |
| ***Macronutrients*** |  |  |  | |  |  | |  |  |  |
| Carbohydrate - total (g) | 190.766 | 90.561 | 223.600 | | 172.888 | -0.944 | | 55 | 0.349 | -0.262 |
| Carbohydrate sugars - total (g) | 90.284 | 91.971 | 50.542 | | 86.002 | -0.093 | | 55 | 0.926 | -0.026 |
| Carbohydrate - fructose (g) | 17.558 | 18.339 | 11.123 | | 9.767 | -0.264 | | 55 | 0.793 | -0.073 |
| Carbohydrate - galactose (g) | 0.650 | 0.049 | 1.051 | | 0.171 | 2.529 | | 55 | 0.014 | 0.702 |
| Carbohydrate -glucose (g) | 17.286 | 18.117 | 11.057 | | 12.305 | -0.260 | | 55 | 0.796 | -0.072 |
| Carbohydrate -lactose (g) | 11.940 | 5.097 | 6.885 | | 7.514 | 3.469 | | 55 | 0.001 | 0.963 |
| Carbohydrate -maltose (g) | 2.572 | 2.809 | 1.669 | | 3.438 | -0.351 | | 55 | 0.727 | -0.097 |
| Carbohydrate -sucrose (g) | 38.772 | 25.130 | 45.854 | | 53.692 | -0.680 | | 55 | 0.500 | -0.189 |
| Carbohydrate -starch (g) | 95.853 | 46.334 | 127.099 | | 87.575 | -1.768 | | 55 | 0.083 | -0.491 |
| Protein (g) | 50.580 | 21.477 | 54.476 | | 33.944 | -0.531 | | 55 | 0.598 | -0.147 |
| Fat - total (g) | 61.443 | 30.880 | 69.892 | | 69.315 | -0.637 | | 55 | 0.527 | -0.177 |
| MUFA - total (g) | 21.362 | 11.089 | 28.464 | | 25.786 | -1.453 | | 55 | 0.152 | -0.403 |
| PUFA - total (g) | 11.228 | 5.330 | 16.219 | | 10.731 | -2.354 | | 55 | 0.022 | -0.653 |
| SFA - total (g) | 23.678 | 12.888 | 19.720 | | 27.703 | 0.738 | | 55 | 0.464 | 0.205 |
| ***Englyst Fibre (g)*** | 15.457 | 18.926 | 6.661 | | 7.705 | -1.775 | | 55 | 0.081 | -0.493 |
| ***Cholesterol (mg)*** | 157.434 | 99.432 | 86.704 | | 230.473 | 1.618 | | 55 | 0.111 | 0.449 |
| ***Micronutrients*** |  |  |  | |  |  | |  |  |  |
| Vit A - retinol (mcg) | 296.527 | 193.737 | 158.167 | | 356.610 | 1.905 | | 55 | 0.062 | 0.529 |
| Vit A - retinol equiv. (mcg) | 940.619 | 528.120 | 898.311 | | 577.045 | 0.279 | | 55 | 0.781 | 0.078 |
| Carotene total (mcg) | 3835.785 | 2329.035 | 4375.868 | | 3181.791 | -0.733 | | 55 | 0.467 | -0.203 |
| Alpha carotene (mcg) | 358.601 | 300.455 | 401.195 | | 352.477 | -0.481 | | 55 | 0.633 | -0.133 |
| Beta carotene (mcg) | 3483.426 | 2150.919 | 4019.390 | | 3053.889 | -0.772 | | 55 | 0.443 | -0.214 |
| Vit B1 - thiamine (mg) | 1.394 | 0.647 | 1.882 | | 0.823 | -2.463 | | 55 | 0.017 | -0.684 |
| Vit B2 - riboflavin (mg) | 1.533 | 1.317 | 0.540 | | 0.741 | 1.260 | | 55 | 0.213 | 0.350 |
| Vit B3- niacin (mg) | 13.131 | 15.940 | 5.118 | | 8.118 | -1.602 | | 55 | 0.115 | -0.445 |
| Vit B6 - pyridoxine (mg) | 1.535 | 0.574 | 1.824 | | 0.814 | -1.560 | | 55 | 0.124 | -0.433 |
| Vit B9 - folate (mcg) | 263.165 | 104.164 | 291.937 | | 132.919 | -0.902 | | 55 | 0.371 | -0.250 |
| Vit B12 - cobalamin (mcg) | 2.093 | 1.096 | 0.967 | | 2.236 | 2.560 | | 55 | 0.013 | 0.710 |
| Vit C - ascorbic acid (mg) | 97.901 | 45.357 | 98.505 | | 58.706 | -0.043 | | 55 | 0.966 | -0.012 |
| Vit D - ergocalciferol (mcg) | 1.105 | 0.796 | 0.811 | | 2.449 | 0.672 | | 55 | 0.505 | 0.186 |
| Vit E - alpha tocopherol equiv. (mg) | 10.881 | 5.388 | 15.075 | | 8.160 | -2.332 | | 55 | 0.023 | -0.647 |
| Calcium (mg) | 888.763 | 763.585 | 398.156 | | 516.085 | 1.019 | | 55 | 0.312 | 0.283 |
| Chloride (mg) | 3243.968 | 1508.415 | 3565.506 | | 2550.671 | -0.599 | | 55 | 0.551 | -0.166 |
| Copper (mg) | 0.965 | 0.409 | 1.476 | | 0.695 | -3.505 | | 55 | 0.001 | -0.973 |
| Iron (mg) | 9.215 | 3.886 | 11.567 | | 5.223 | -1.929 | | 55 | 0.059 | -0.535 |
| Iodine (mcg) | 90.886 | 45.266 | 67.857 | | 103.788 | 1.166 | | 55 | 0.249 | 0.324 |
| Potassium (mg) | 2825.182 | 1091.714 | 3224.357 | | 1272.201 | -1.243 | | 55 | 0.219 | -0.345 |
| Magnesium | 275.142 | 101.645 | 344.785 | | 145.982 | -2.111 | | 55 | 0.039 | -0.586 |
| Manganese | 2.910 | 1.166 | 4.407 | | 1.958 | -3.624 | | 55 | 0.001 | -1.006 |
| Phosphorus (mg) | 1040.739 | 412.630 | 1040.934 | | 628.223 | -0.001 | | 55 | 0.999 | -0.174 |
| Selenium (mcg) | 31.590 | 14.210 | 38.231 | | 26.136 | -1.247 | | 55 | 0.218 | 0.000 |
| Sodium (mg) | 2159.151 | 2392.467 | 1018.367 | | 1792.209 | -0.629 | | 55 | 0.532 | -0.346 |
| Zinc (mg) | 6.169 | 6.965 | 2.424 | | 3.685 | -0.983 | | 55 | 0.330 | -0.273 |

## **Supplementary Discussion: Differences between vegans and vegetarians**

It is important to note that a key outcome of the present results is the significant differences found between vegans and vegetarians when it came to dietary identity. Specifically, vegans demonstrated significantly higher scores on private regard, prosocial, personal, and moral motivation. On the other hand, vegetarians reported higher scores on public and outgroup regard as well as dietary strictness. Our results are consistent and support the limited but growing evidence that vegans have more positive feelings towards other vegans (private regard), more negative feelings towards both being judged by others (public regard) and by judging non vegans (outgroup regard) and stronger motivations (prosocial, personal and moral) in comparison to vegetarians on their dietary choice (Kirsten et al., 2020; Paulus et al., 2016; Rosenfeld & Burrow, 2018; Rosenfeld, 2019; Senra et al., 2016).

To our knowledge, ours is the first research to demonstrate that dietary strictness was higher in vegetarians than vegans. This is in contrast with past research that has shown that dietary strictness did not differ between these two groups (Rosenfeld, 2019; Rothgerber, 2015). One possibility for this discrepancy is that previous research has used participants who self-labelled as vegetarian but ate fish and poultry (Rosenfeld, 2019). In the present research we ensured that self-identification of vegetarianism and veganism was consistent with dietary profile (i.e., vegetarians avoiding meat, fish and poultry but eating eggs and dairy, vegans avoiding all animal products). At first glance, our results appear counterintuitive given that we demonstrated stronger prosocial, personal and moral motivations in vegans compared to vegetarians which would presumably lead to greater dietary strictness in the former group. One tentative possibility in explaining this finding is that the restrictive nature of the vegan diet reduces the food options available compared to a vegetarian diet and therefore adhering to these restrictions may be more difficult for vegans. There may be other external factors at play such as the desire to fit in during social situations or at work that may impact dietary strictness. Certainly, future research could disentangle these issues more directly to explore differences in dietary adherence between vegans and vegetarians.

### Dietary Intakes

One of the key questions in the present research is whether plant-based diets are linked to mental and sleep health. Previous research has demonstrated that poor diet quality is associated with poor mental and sleep health (Hepsomali & Groeger, 2021) and that plant-based diets are linked to enhanced diet quality (Allès et al., 2017; Clarys et al., 2014; Hargreaves et al., 2020). However, there is conflicting research as to whether plant-based diets are linked to improved mental and sleep health outcomes and the mixture rather than separation of different plant-based diets within this research (i.e., vegans, vegetarians) might be contributing to the lack of a clear picture. Our research therefore addressed the question as to whether dietary intake *between* plant-based diets differed and whether such differences are linked to any psychological and behavioural characteristics that might predict mental and sleep health. Overall, we found key dietary intake differences between vegans and vegetarians but no associated differences in mental or sleep wellbeing. The results are discussed in more depth below.

Our results demonstrated that vegans consumed significantly less lactose and Vit B12 compared to vegetarians. As the vegan diet excludes all animal products including those containing lactose such as milk, this difference in lactose consumption is unsurprising. There is very little research to date that has explored the effects of lactose on mental or sleep health, but a recent study demonstrated that low lactose intake, whilst being associated with lower physical quality of life, had no impact on mental or social life quality (Garipoğlu et al., 2022).. This is consistent with our findings demonstrating that there were no differences in mental wellbeing, depression, anxiety or indeed stress between vegans and vegetarians in the present research, despite the former consuming significantly less lactose.

Compared to omnivores, research has often reported Vit B12 deficiencies in those following a plant-based diet (see (Dimitra et al., 2021)for a review) but to our knowledge, our research is the first to demonstrate that such deficiencies are greater in vegans than in vegetarians. Vit B12 is found in foods such as meat, fish, poultry, eggs, and dairy products all of which are restricted in vegan diets and substantially reduced in vegetarian diets which is consistent with our current results. With regards to mental health, research has demonstrated that enhanced levels of Vit B12 lead to improved outcomes for those suffering from depressive disorders (Hintikka et al., 2003; Sangle et al., 2020). Past research has also shown a link between vegetarians and depression (Meesters et al., 2017), however this research neither tested vegans nor assessed Vit B12 intake. More recent research that explored brain volume, diet and depression between vegetarians and non-vegetarians, found the former to have lower levels of Vit B12 and higher levels of depression but no effects on brain volume (Berkins et al., 2021). Whilst past research therefore suggests a link between Vit B12, vegetarianism and depression, our results do not support this relationship as despite significantly lower levels of Vit B12 in vegans compared to vegetarians, our research showed there was no related difference between these groups in mental wellbeing, depression, anxiety, stress, or sleep health. One possibility for this discrepancy is that ours is the first research to clearly separate participants into different plant-based diets ensuring that nutritional intake is consistent with dietary identification (i.e., ensuring that participants identifying themselves as vegan do not consume any animal products). Given the clear significant differences in Vit B12 consumption between vegans and vegetarians we demonstrated, such a strict protocol is necessary to properly investigate any effects of diet on mental and sleep health. In light of previous research, our results would suggest that the key linking factor with depression is not diet-type per se, but rather Vit B12 deficiency. Future research will need to disentangle this issue further employing larger population samples given the pilot nature of the present research.

Our results also demonstrated that vegans consumed more galactose and polyunsaturated fatty acid (PUFA) compared to vegetarians, suggesting a higher intake of nuts, seeds and fruit in the former group. Intake of Vitamin B1, Vitamin E, copper, magnesium, and manganese was also higher in vegans, and this might be related to the higher use of supplementation of vitamins and minerals that this group tend to have than other diet types including vegetarians (Kirk et al., 1999).

Overall, our dietary intake results therefore support past research showing that plant-based diets are not linked to mental or sleep health outcomes (Sengul, 2022) and reveal that such null effects are not due to lack of differentiation between plant-based diets nor to Vit B12 deficiencies. Whilst our results are not consistent with research showing a link between diet and improved mental (Firth et al., 2019; Hepsomali & Groeger, 2021) and sleep health (Crawford et al., 2017), this research was based on plant-forward diets which do contain low levels of both meat and fish consumption. This further demonstrates the importance and need to separate vegans and vegetarians when exploring any relationship between diet and mental and sleep wellbeing.

### Questionnaire outcomes

A key outcome of the present results is the significant differences found between vegans and vegetarians when it came to dietary identity. Specifically, vegans demonstrated significantly higher scores on private regard, prosocial, personal, and moral motivation. On the other hand, vegetarians reported higher scores on public and outgroup regard as well as dietary strictness. Our results are consistent and support the limited but growing evidence that vegans have more positive feelings towards other vegans (private regard), more negative feelings towards both being judged by others (public regard) and by judging non vegans (outgroup regard) and stronger motivations (prosocial, personal, and moral) in comparison to vegetarians on their dietary choice (Kirsten et al., 2020; Rosenfeld & Burrow, 2018) .

To our knowledge, ours is the first research to demonstrate that dietary strictness was higher in vegetarians than vegans. This is in contrast with past research that has shown that dietary strictness did not differ between these two groups (Rosenfeld, 2019; Rothgerber, 2015). One possibility for this discrepancy is that previous research has used participants who self-labelled as vegetarian but ate fish and poultry (Rosenfeld, 2019). In the present research we ensured that self-identification of vegetarianism and veganism was consistent with dietary profile (i.e., vegetarians avoiding meat, fish and poultry but eating eggs and dairy, vegans avoiding all animal products). At first glance, our results appear counterintuitive given that we demonstrated stronger prosocial, personal, and moral motivations in vegans compared to vegetarians which would presumably lead to greater dietary strictness in the former group. One tentative possibility in explaining this finding is that the restrictive nature of the vegan diet reduces the food options available compared to a vegetarian diet and therefore adhering to these restrictions may be more difficult for vegans. There may be other external factors at play such as the desire to fit in during social situations or at work that may impact dietary strictness. Certainly, future research could disentangle these issues more directly to explore differences in dietary adherence between vegans and vegetarians.

Our results demonstrated that vegans and vegetarians did not differ on BFI, EQ, DASS, WEMWBS, and PSQI measures. This is in contrast with previous research showing higher levels of neuroticism (as measured within the BFI) in vegetarians compared to vegans (Müssig et al., 2022) and higher levels of openness and empathy (Kessler et al., 2016). It is unclear why these differences have occurred although the effects in previous research were relatively small, and the research carried out in a different country to the present research, and it is therefore unclear what impact (if any) societal differences may have had on these effects.

# References

Allès, B., Baudry, J., Méjean, C., Touvier, M., Péneau, S., Hercberg, S., & Kesse-Guyot, E. (2017). Comparison of Sociodemographic and Nutritional Characteristics between Self-Reported Vegetarians, Vegans, and Meat-Eaters from the NutriNet-Santé Study. *Nutrients, 9*(9), 1023-18. <https://doi.org/10.3390/nu9091023>

Beezhold, B. L., & Johnston, C. S. (2012). Restriction of meat, fish, and poultry in omnivores improves mood: A pilot randomized controlled trial. *Nutrition Journal, 11*(1), 9. <https://doi.org/10.1186/1475-2891-11-9>

Berkins, S., Schiöth, H. B., & Rukh, G. (2021). Depression and Vegetarians: Association between Dietary Vitamin B6, B12 and Folate Intake and Global and Subcortical Brain Volumes. *Nutrients, 13*(6), 1790. doi: 10.3390/nu13061790. <https://doi.org/10.3390/nu13061790> [doi]

Clarys, P., Deliens, T., Huybrechts, I., Deriemaeker, P., Vanaelst, B., De Keyzer, W., Hebbelinck, M., & Mullie, P. (2014). Comparison of Nutritional Quality of the Vegan, Vegetarian, Semi-Vegetarian, Pesco-Vegetarian and Omnivorous Diet. *Nutrients, 6*(3), 1318-1332. <https://doi.org/10.3390/nu6031318>

Crawford, A., Aggarwal, B., Greenberger, H., M., Liao, M., & St-Onge, M. (2017). Association of plant-based protein with sleep quality and duration in women. *Society of General Internal Medicine Annual Meeting. J GEN INTERN MED, 32 (Suppl 2)*, 83-808. <https://doi.org/10.1007/s11606-017-4028-8>

Dimitra, R., Bakaloudi, Halloran, A., Rippin, H. L., Oikonomidou, C., Dardavesis, T. I., Williams, J., Wickramasinghe, K., Breda, J., & Chourdakis, M. (2021). Intake and adequacy of the vegan diet. A systematic review of the evidence. *Clinical Nutrition, 40*, 3503-3521. <https://doi.org/10.1016/j.clnu.2020.11.035>

Firth, J., Marx, W., Dash, S., Carney, R., Teasdale, S. B., Solmi, M., Stubbs, B., Schuch, F. B., Carvalho, A. F., Jacka, F., & Sarris, J. (2019). The Effects of Dietary Improvement on Symptoms of Depression and Anxiety: A Meta-Analysis of Randomized Controlled Trials. *Psychosomatic Medicine, 81*(3), 265-280. <https://doi.org/10.1097/PSY.0000000000000673>

Foster, M., Chu, A., Petocz, P., & Samman, S. (2013). Effect of vegetarian diets on zinc status: a systematic review and meta-analysis of studies in humans. *Journal of the Science of Food and Agriculture, 93*(10), 2362-2371. <https://doi.org/10.1002/jsfa.6179>

Gajski, G., Gerić, M., Vučić Lovrenčić, M., Božičević, S., Rubelj, I., Nanić, L., Škrobot Vidaček, N., Bendix, L., Peraica, M., Rašić, D., Domijan, A., Gluščić, V., Jurasović, J., Orct, T., Cvijetić Avdagić, S., Jurak, G., Bošnir, J., & Garaj-Vrhovac, V. (2018). Analysis of health-related biomarkers between vegetarians and non-vegetarians: A multi-biomarker approach. *Journal of Functional Foods, 48*, 643-653. <https://doi.org/10.1016/j.jff.2018.07.054>

Gallego-Narbón, A., Zapatera, B., Barrios, L., & Vaquero, M. P. (2019). Vitamin B12 and folate status in Spanish lacto-ovo vegetarians and vegans. *Journal of Nutritional Science, 8*, e7. <https://doi.org/10.1017/jns.2019.2>

Garipoğlu, G., Ersoy, N., Gülşen, M., & Özgürtaş, T. (2022). Effect of lactose intolerance severity on food intake and quality of life in adults with lactose intolerance in Turkey. *Journal of Health Research, 36*(3), 533-540. <https://doi.org/10.1108/JHR-12-2020-0617>

Gilsing, A. M. J., Crowe, F. L., Lloyd-Wright, Z., Sanders, T. A. B., Appleby, P. N., Allen, N. E., & Key, T. J. (2010). Serum concentrations of vitamin B12 and folate in British male omnivores, vegetarians and vegans: results from a cross-sectional analysis of the EPIC-Oxford cohort study. *European Journal of Clinical Nutrition, 64*(9), 933-939. <https://doi.org/10.1038/ejcn.2010.142>

Godos, J., Ferri, R., Caraci, F., Cosentino, F. I. I., Castellano, S., Galvano, F., & Grosso, G. (2019). Adherence to the Mediterranean Diet is Associated with Better Sleep Quality in Italian Adults. *Nutrients, 11*(5), 976. <https://doi.org/10.3390/nu11050976>

Hargreaves, S. M., Araujo, W. M. C., Nakano, E. Y., & Zandonadi, R. P. (2020). Brazilian vegetarians diet quality markers and comparison with the general population: A nationwide cross-sectional study. *PloS One, 15*(5), e0232954. <https://doi.org/10.1371/journal.pone.0232954>

Hepsomali, P., & Groeger, J. A. (2021). Diet, Sleep, and Mental Health: Insights from the UK Biobank Study. *Nutrients, 13*(8), 2573. <https://doi.org/10.3390/nu13082573>

Hintikka, J., Tolmunen, T., Tanskanen, A., & Viinamäki, H. (2003). High vitamin B12 level and good treatment outcome may be associated in major depressive disorder. *BMC Psychiatry, 3*(1), 17. <https://doi.org/10.1186/1471-244X-3-17>

Iguacel, I., Huybrechts, I., Moreno, L. A., & Michels, N. (2021). Vegetarianism and veganism compared with mental health and cognitive outcomes: a systematic review and meta-analysis. *Nutrition Reviews, 79*(4), 361-381. <https://doi.org/10.1093/nutrit/nuaa030>

Jacka, F. N., Pasco, J. A., Mykletun, A., Williams, L. J., Hodge, A. M., O'Reilly, S. L., Nicholson, G. C., Kotowicz, M. A., & Berk, M. (2010). Association of Western and Traditional Diets With Depression and Anxiety in Women. *The American Journal of Psychiatry, 167*(3), 305-311. <https://doi.org/10.1176/appi.ajp.2009.09060881>

Jain, R., Larsuphrom, P., Degremont, A., Latunde‐Dada, G. O., & Philippou, E. (2022). Association between vegetarian and vegan diets and depression: A systematic review. *Nutrition Bulletin, 47*(1), 27-49. <https://doi.org/10.1111/nbu.12540>

Kapoor, A., Baig, M., Tunio, S. A., Memon, A. S., & Karmani, H. (2017). Neuropsychiatric and neurological problems among Vitamin B12 deficient young vegetarians. *Neurosciences, 22*(3), 228-232. <https://doi.org/10.17712/nsj.2017.3.20160445>

Kessler, C. S., Holler, S., Joy, S., Dhruva, A., Michalsen, A., Dobos, G., & Cramer, H. (2016). Personality Profiles, Values and Empathy: Differences between Lacto-Ovo-Vegetarians and Vegans. *Complementary Medicine Research, 23*(2), 95-102. <https://doi.org/10.1159/000445369>

Kirk, S. F., Cade, J. E., Barrett, J. H., & Conner, M. (1999). Diet and lifestyle characteristics associated with dietary supplement use in women. *Public Health Nutrition, 2*(1), 69-73. <https://doi.org/10.1017/S1368980099000099>

Kirsten, H., Seib-Pfeifer, L., Lüth, C. A., & Rosenfeld, D. L. (2020). Validation and application of a German version of the Dietarian Identity Questionnaire: Revealing differences between omnivores, vegetarians, and vegans. *Food Quality and Preference, 86*, 103988. <https://doi.org/10.1016/j.foodqual.2020.103988>

Larsson, C. L., & Johansson, G. K. (2002). Dietary intake and nutritional status of young vegans and omnivores in Sweden. *The American Journal of Clinical Nutrition, 76*(1), 100-106. <https://doi.org/10.1093/ajcn/76.1.100>

Meesters, A. N. R., Maukonen, M., Partonen, T., Männistö, S., Gordijn, M. C. M., & Meesters, Y. (2017). Is There a Relationship between Vegetarianism and Seasonal Affective Disorder? A Pilot Study. *Neuropsychobiology, 74*(4), 202-206. <https://doi.org/10.1159/000477247>

Müssig, M., Pfeiler, T. M., & Egloff, B. (2022). Minor and inconsistent differences in Big Five personality traits between vegetarians and vegans. *PloS One, 17*(6), e0268896. <https://doi.org/10.1371/journal.pone.0268896>

Neufingerl, N., & Eilander, A. (2021). Nutrient Intake and Status in Adults Consuming Plant-Based Diets Compared to Meat-Eaters: A Systematic Review. *Nutrients, 14*(1), 29. <https://doi.org/10.3390/nu14010029>

Paulus, D. J., Vanwoerden, S., Norton, P. J., & Sharp, C. (2016). From neuroticism to anxiety: Examining unique contributions of three transdiagnostic vulnerability factors. *Personality and Individual Differences, 94*, 38-43. <https://doi.org/10.1016/j.paid.2016.01.012>

Rosenfeld, D. L. (2019). A comparison of dietarian identity profiles between vegetarians and vegans. *Food Quality and Preference, 72*, 40-44. <https://doi.org/10.1016/j.foodqual.2018.09.008>

Rosenfeld, D. L., & Burrow, A. L. (2018). Development and validation of the Dietarian Identity Questionnaire: Assessing self-perceptions of animal-product consumption. *Appetite, 127*, 182-194. <https://doi.org/10.1016/j.appet.2018.05.003>

Rothgerber, H. (2015). Can you have your meat and eat it too? Conscientious omnivores, vegetarians, and adherence to diet. *Appetite, 84*, 196-203. <https://doi.org/10.1016/j.appet.2014.10.012>

Sakkas, H., Bozidis, P., Touzios, C., Kolios, D., Athanasiou, G., Athanasopoulou, E., Gerou, I., & Gartzonika, C. (2020). Nutritional Status and the Influence of the Vegan Diet on the Gut Microbiota and Human Health. *Medicina, 56*(2), 88. <https://doi.org/10.3390/medicina56020088>

Sangle, P., Sandhu, O., Aftab, Z., Anthony, A. T., & Khan, S. (2020). Vitamin B12 Supplementation: Preventing Onset and Improving Prognosis of Depression. *Cureus, 12*(10), e11169. <https://doi.org/10.7759/cureus.11169> [doi]

Schüpbach, R., Wegmüller, R., Berguerand, C., Bui, M., & Herter-Aeberli, I. (2015). Micronutrient status and intake in omnivores, vegetarians and vegans in Switzerland. *European Journal of Nutrition, 56*(1), 283-293. <https://doi.org/10.1007/s00394-015-1079-7>

Sengul, P. (2022). Comparison of vegan and non-vegan diets on memory and sleep quality. *Clinical Nutrition Open Science, 43*, 78-84. <https://doi.org/10.1016/j.nutos.2022.05.005>

Senra, C., Merino, H., & Ferreiro, F. (2016). Are Worry and Rumination Specific Pathways Linking Neuroticism and Symptoms of Anxiety and Depression in Patients with Generalized Anxiety Disorder, Major Depressive Disorder and Mixed Anxiety-Depressive Disorder? *PLoS ONE, 11*(5)<https://doi.org/10.6084/m9.figshare.3385756>

Weikert, C., Trefflich, I., Menzel, J., Obeid, R., Longree, A., Dierkes, J., Meyer, K., Herter-Aeberli, I., Mai, K., Stangl, G. I., Müller, S. M., Schwerdtle, T., Lampen, A., & Abraham, K. (2020). Vitamin and Mineral Status in a Vegan Diet. *Deutsches Ärzteblatt International, 117*(35-36), 575-582. <https://doi.org/10.3238/arztebl.2020.0575>

stylefix
